# The effect of high tibial osteotomy on the results of total knee arthroplasty: a matched case control study

**DOI:** 10.1186/1471-2474-8-74

**Published:** 2007-08-03

**Authors:** Tom M van Raaij, Wouter Bakker, Max Reijman, Jan AN Verhaar

**Affiliations:** 1 Department of Orthopaedics, Erasmus University Medical Centre, Rotterdam, the Netherlands

## Abstract

**Background:**

We performed a matched case control study to assess the effect of prior high tibia valgus producing osteotomy on results and complications of total knee arthroplasty (TKA).

**Methods:**

From 1996 until 2003 356 patients underwent all cemented primary total knee replacement in our institution. Twelve patients with a history of 14 HTO were identified and matched to a control group of 12 patients with 14 primary TKA without previous HTO. The match was made for gender, age, date of surgery, body mass index, aetiology and type of prosthesis. Clinical and radiographic outcome were evaluated after a median duration of follow-up of 3.7 years (minimum, 2.3 years). The SPSS program was used for statistical analyses.

**Results:**

The index group had more perioperative blood loss and exposure difficulties with one tibial tuberosity osteotomy and three patients with lateral retinacular releases. No such procedures were needed in the control group. Mid-term HSS, KSS and WOMAC scores were less favourable for the index group, but these differences were not significant. The tibial slope of patients with prior HTO was significantly decreased after this procedure. The tibial posterior inclination angle was corrected during knee replacement but posterior inclination was significantly less compared to the control group. No deep infection or knee component loosening were seen in the group with prior HTO.

**Conclusion:**

We conclude that TKA after HTO seems to be technically more demanding than a primary knee arthroplasty, but clinical outcome was almost identical to a matched group that had no HTO previously.

## Background

Medial unicompartmental osteoarthritis of the knee is a common clinical problem. In the active patient with a life expectancy of 20 years or more a high tibia valgus osteotomy (HTO) is a generally accepted treatment that can result in excellent pain relief and function improvement. However results seem to deteriorate in time and an overall failure rate of 24% at 10 years has been reported [1]. Most likely due to the natural course of unicompartmental osteoarthritis progression of symptoms occurs and this group of patients may require knee replacement.

Difficult exposure of the tibia due to soft tissue scaring, patellar mechanism eversion difficulties, reduction of the amount of tibia bone stock, tibia plateau tilting, retained osteosynthesis and subacute infection are technical points to be dealt with when performing a total knee arthroplasty (TKA) after proximal tibia osteotomy [2]. Another important factor influencing the outcome of TKA after HTO is patient selection [3]. Patient cohort disparity may therefore be one of the causes that some report substandard total knee arthroplasty outcome after a high tibial osteotomy [4-6] while others see no clinical or radiographic difference for TKA with or without an osteotomy [7,8].

Randomized controlled trials (RCTs) are considered the ideal and highest level of evidenced based medicine [9]. However, an RCT may not be an appropriate standard of evidence for evaluating most surgical treatments. Very few operations can be randomized for ethical, scientific, or practical reasons. Numerous "good" surgical practices have evolved into "standard of care" without being randomized against placebo or ineffective treatment options [10]. An RCT on the effect of previous HTO on TKA outcome would hardly be ethical. The young, active patient with symptomatic medial compartmental osteoarthritis would be denied of standard operative care by not performing tibial osteotomy as a placebo-control arm. Thus the highest practicable level of evidence will be a representative observational study [10].

We conducted a case control study to assess the influence of high tibia valgus osteotomy on results and complications of total knee replacement. In the aim to prevent cohort disparity we matched for diagnosis, time of follow-up, body weight and significant risk factors for failure of TKA [11] to select an ideal comparison group.

## Methods

Between January 1996 and June 2003, a series of 356 all cemented primary total knee arthroplasties (TKA) was performed in the author's institution. Sixteen patients with a history of 18 proximal tibial osteotomies prior to total knee replacement were identified. Two patients had died and two patients were lost to follow up.

The index group was compromised of 14 knees in 10 women and 2 men who underwent total knee replacement 4.8 (range 1.3 – 8.9) years after HTO. A lateral closing wedge technique through a transverse incision with the patient in supine position was performed in all patients. The osteotomy was fixated with two step staples. After surgery a standard cylinder plaster cast was applied for 6 weeks. All patients were mobilised on the first postoperative day, and partial weight-bearing with the use of two crutches was allowed for 6 weeks. After bony healing the staples were removed in all knees except one; in 9 knees before, in 3 during, and in one knee after joint replacement. Symptomatic medial compartment osteoarthritis was diagnosed in all cases. The median age at the time of total knee surgery was 60 (range 51 – 75) years and the median body mass index (BMI) of this group was 31.3 (range 26.2 – 41.5) kg/m^2^. Nine patients had surgery prior to tibial osteotomy, including four who had meniscectomy, one who had arthroscopy with debridement, one who had a cartilage transplantation, one who had a failed valgus closing wedge tibia osteotomy performed in another institution, and two who had surgery because of a fracture around the knee.

This group was matched with a control group of 14 primary total knees in 12 patients selected from the same cohort of patients with total knee replacement. The match was made for gender, age, date of surgery, BMI, aetiology and type of prosthesis (Table 1). Ten patients in the control group underwent prior surgery before knee replacement. Six patients had a meniscectomy, two had surgery because of a fracture around the knee, one had a diagnostic arthroscopy to evaluate posterior cruciate ligament insufficiency and one had a peroneal nerve release. The indication for TKA was symptomatic osteoarthritis in all patients.

**Table 1:** Demographic data for 14 knees with a closing wedge high tibia osteotomy prior to TKA (index group) and 14 knees with a primary TKA (control group)

Demographic	Index	Control
Gender, female : male	10 : 2	10 : 2
Age, median years	60 (51 – 75)	61 (51 – 75)
Follow-up, median years	3.7 (2.3 – 8.8)	4.0 (2.3 – 8.3)
BMI, median kg/m^2^	31.1 (26.2 – 41.5)	28.6 (24.5 – 37.3)
ASA		
1	1	2
2	10	10
3	3	2
OA, Kellgren-Lawrence score		
Grade 2	1	1
Grade 3	12	9
Grade 4	1	4
OA, Ahlbäck score		
Grade 1	4	4
Grade 2	6	8
Grade 3	3	1
Grade 4	1	1
Prosthesis, Kinemax : Genesis	11 : 2	10 : 3

Two types of cruciate retaining all cemented total knee prosthesis (Kinemax, Howmedica International Inc, Co. Clare, Ireland and Genesis II, Smith & Nephew, Memphis, U.S.A.) were used in both groups. One patient in the index group needed a posteriorly stabilized knee prosthesis and was matched to a patient in the control group with the same type of prosthesis.

Patients in both groups underwent clinical and radiographic evaluation at a minimum follow up of two years. Pre- and postoperative data of knee range of motion were available for all patients. Knee pain was measured by a visual analogue scale (VAS (0 – 10)) [12]. Knee function was evaluated by the Hospital for Special Surgery Score (HSS), the Knee Society Score (KSS) and the Western Ontario and McMaster University Osteoarthritis Index (WOMAC) [13-15].

Pre- and postoperative radiography of the knee included an anteroposterior radiograph in standing position to measure the femorotibial angle (FTA) [16]. A true lateral radiograph of the knee in at least 30 degrees of flexion was used to determine the length of the patella tendon according to Insall-Salvati (IS ratio) [17]. The posterior inclination angle of the tibia plateau (PI) was measured on a lateral radiograph according to Moore-Harvey [18]. The Kellgren-Lawrence and Ahlbäck scores were used to determine the level of osteoarthritis [19,20]. Fixation of the knee components was evaluated using the Knee Society Roentgenographic Evaluation System [21].

The SPSS program was used for the statistical analyses. A p-value of 0.05 was considered statistically significant. The sample size was calculated based on an expected inferior clinical result for the index group. An inferior result was defined as 15 points difference in HSS score. To detect such a difference with one-sided testing (α = 0.05 and power of 80%) we needed to include 12 patients in each group. The data for this investigation were collected and analyzed in compliance with the procedures and policies set forth by the Helsinki Declaration, and all patients gave their informed consent.

## Results

No significant differences were noted between the two groups with respect for gender, age, time of follow-up, American Society of Anaesthesiologists (ASA) risk score [22] and type of knee prosthesis ratio (Table 1). Both groups showed overweighted patients with a higher median BMI for the index group; this was, however, not significantly different from the control group. The different levels of osteoarthritis before knee replacement were equally distributed in both groups using the Kellgren-Lawrence and Ahlading systems (Table 1).

All total knees in both groups were approached by the standard medial parapatellar incision.

The median operative time in the index group was not significantly different from the control group (p = 0.173) with 120 (range 95 – 165) minutes compared to 115 (range 90 – 135) minutes; respectively. The patient group with a previous osteotomy suffered from more perioperative blood loss with a median of 450 (range 100 – 915) ml compared to a median blood loss of 225 (range 100 – 600) ml in the control group; this difference was not significant (p = 0.071). The index group required one tibial tuberosity osteotomy and three lateral retinaculum releases. The control group needed no tuberosity osteotomy or lateral releases. There was a trend (p = 0.092) towards the use of a thicker polyethylene tibial component composite in the index group, with a mean thickness of 10 (range 8 – 18) mm compared with a median of 8 (range 8 – 12) mm in the control group.

### Postoperative complications

No infections were seen in both groups postoperatively. Two patients, one in each group, were mobilized under anaesthesia because of limited range of motion.

### Clinical and radiological outcome

The range of motion postoperative showed no significant difference between both groups (Table 2). The postoperative knee flexion in the index and control group had a median of 110° (range 95 – 130°) and 120° (range 80 – 130°), respectively. The postoperative median VAS score was 4.5 (range 0 – 9) in the index group and 3.7 (range 0 – 9) in the control group; this difference was not significant. The functional outcome analysed by the HSS, KSS and WOMAC scores is presented in Table 3. Although all scores were worse for the index group no significant differences were noted.

**Table 2:** Range of motion in both groups before and after TKA

		Index*	Control*	Significance
Flexion	Pre TKA	100° (60 – 150)	120° (90 – 130)	NS
	Post TKA	110° (95 – 130)	120° (80 – 130)	NS
Extension	Pre TKA	0° (-15 – 0)	0° (-40 – 0)	NS
	Post TKA	0° (-10 – 5)	0° (-20 – 10)	NS

* The values are given as the median (range)

- Negative value indicates extension deficit

**Table 3:** Functional outcome in both groups after TKA

		Index*	Control*	Significance
Score	[0–100]			
HSS		78.5 (48 – 91)	82 (57 – 95)	NS
				
KSS	Knee	79 (45 – 105)	90 (45 – 110)	NS
	Function	70 (10 – 90)	80 (20 – 100)	NS
				
WOMAC	Pain	57.5 (30 – 100)	80 (20 – 100)	NS
	Stiffnes	43.8 (12.5 – 100)	62.5 (12.5 – 100)	NS
	Function	56.6 (17 – 100)	66.1 (14 – 100)	NS

* The values are given as the median (range)

The radiographic results for both groups are given in Table 4 and 5. The index group had a significant (p < 0.0001) increase of the median FTA from – 3° (range -9 – 0°) before HTO to 3° (range -2 – 9°) before TKA. The PI (p = 0.04) and the IS ratio (p = 0.03) were both significantly decreased after HTO from 6° (range -2 to 14°) to 3° (range -13 to 16°) and from 1.06 (range 0.74 – 1.29) to 0.95 (range 0.58 – 1.20), respectively.

**Table 4:** Radiological outcome in both groups before TKA

	Index*			Index*	Control*	
	pre HTO	pre TKA	P	pre TKA	pre TKA	P
FTA^a^	-3.0° (-9; 0)	3.0° (-2; 9)	< 0.0001	3.0° (-2; 9)	2.0° (-3; 11)	NS
PI^b^	6.0° (-2; 14)	3.0° (-13; 16)	0.04	3.0° (-13; 16)	6.5° (4; 16)	0.04
IS^c^	1.06 (0.74; 1.29)	0.95 (0.58; 1.20)	0.002	0.95 (0.58; 1.20)	1.07 (0.76; 1.29)	0.05

* The values are given as the mean (range)

- Negative value indicates varus alignment

^a ^Femorotibial angle

^b ^Posterior inclination tibia plateau

^c ^Patella height: Insall-Salvati ratio

**Table 5:** Radiological outcome in both groups after TKA

		Index*	Control*	Significance
Alignment^a^		5.0° (-4 – 14)	6.0° (-4 – 9)	NS
Femoral component^b^		2.0° (-3 – 8)	2.0° (-5 – 8)	NS
Tibial component^c^		7.0° (0 – 12)	12.0° (2 – 20)	p = 0.026
Insall – Salvati ratio		1.25 (0.90 – 1.79)	1.10 (0.90 – 1.46)	NS
Loosening^d^	Femoral (mm)	N = 1 (3)	N = 2 (2 + 3)	NS
	Tibial (mm)	0	N = 1 (9)	NS
	Patella (mm)	0	0	NS

* The values are given as the median (range)

- Negative value indicates varus alignment

^a ^Femorotibial angle (FTA)

^b ^Flexion angle femoral component

^c ^Posterior inclination tibial component

^d ^Loosening according to the Knee Society Roentgenographic Evaluation System

Subsequently there were significant differences between the index and control groups according to the preoperative PI (3° (range -13 to 16°) versus 6.5° (range 4 to 16°)) and preoperative IS ratio (0.95 (range 0.58 – 1.20) versus 1.07 (range 0.76 – 1.29)).

Postoperative, the two groups showed no significant differences for anteroposterior alignment, femoral component placement and patellar height. The tibial component was placed with significantly (p = 0.025) less posterior slope in the index group, respectively 7° (range 0 to 12°) and 12° (range 2 to 20°) in the control group. Both groups had an increase of patella height postoperatively, which was only significant for the study group (p = 0.002).

The Knee Society Roentgenographic Evaluation System revealed one patient with a numeric score of 3 for the femoral component in the index group. No radiological tibial or patellar loosening was seen in the index group. The control group had two patients with radiological femoral component loosening with a score of 2 and 3, respectively. Scores of four or less are probably not significant [21].

One patient in the control group showed a numeric score of 9 for the tibial component with malposition. This knee was successfully revised 26 months after total knee replacement. No patellar loosening was seen in the control group.

## Discussion and conclusion

Proximal tibial osteotomy is a well-accepted treatment of medial unicompartmental osteoarthritis of the knee with varus malalignment in young and active patients [1]. In general, however, progression of disease will occur and ultimately many patients require TKA. In the present study osteotomy delayed total knee replacement with a median of 4.8 years. This amount of time bought before performing arthroplasty compares well with other studies [23,24].

Conflicting results of TKA after HTO have been reported. Some studies identify no clinical or radiographic difference in outcome for TKA with or without a previous osteotomy [7,8] while others see substandard TKA outcome after HTO [5,6]. Technical difficulties such as soft tissue scarring, patella infera, limb malalignment, reduced bone stock of the proximal tibia, tibial plateau tilting and retained osteosynthesis material have been recognized to contribute to suboptimal components positioning and soft-tissue balancing [2,3]. Risk of selection bias in non-randomised studies may be another cause of differences in outcome [25]. Patients who had a previous osteotomy are a highly selected population with presumably an unfavourable demographic status [3]. We attempted to minimise selection bias by controlling for known prognostic factors. That is why we conducted a case control study matched for diagnosis, time of follow-up, BMI, gender, age and type of prosthesis. We realise, however, that the present study cannot address the problem of unknown or immeasurable prognostic factors.

In our matched case control study we found less favourable results for a total knee replacement after previous osteotomy compared to a primary knee arthroplasty. Knee replacement after closing wedge tibial osteotomy showed a trend towards prolonged median operative time and a greater amount of blood loss. Hardware removal during knee implant surgery only occurred in three knees, and surgical records showed no difficulties in taking out the step staples. A more plausible reason may have been the significant decline in patellar height (mean IS ratio = 0.93). Patellar tendon shortening, and a decreased distance from the tubercle to the joint after closing wedge osteotomy proximal to the tuberosity, make patellar eversion more difficult [23]. In our series we had to perform three lateral retinacular releases and one tuberosity osteotomy to facilitate eversion of the patella and the patellar ligament. Soft tissue balancing in the valgus knee after closing wedge osteotomy could have been another reason. The trend to use a thicker insert in the study group suggests significantly more lateral dissection.

Our index group developed no flexion contracture post closing wedge osteotomy and the knee range of motion after knee replacement did not differ between the two groups. The alignment considering the femorotibial angle was corrected close to the optimal 6 degrees of valgus in both groups. No significant differences were noted. The femoral component was placed in mild flexion but did not differ between the two groups. The tibial posterior inclination however was significantly decreased after osteotomy (Table 4). We managed to restore the posterior slope during knee replacement but posterior inclination was still significantly less compared to the control group (Table 5). Loss of tibial slope has been described after closing wedge osteotomy and is associated with patella infera. Also a relative elevation of the posterior cruciate ligament can occur leading to PCL insufficiency [26]. In our series we just needed one posterior stabilized knee arthroplasty due to PCL insufficiency. We were able to correct patella infera, and the patellar height after TKA in the index group did not differ significant from the control group. Especially after excluding one patient in the index group with bilateral patella alta after re-insertion of traumatic bilateral patella tendon rupture the patellar ratio (median IS ratio = 1.19) equalled that of the control group (median IS ratio = 1.10).

Literature suggests that after primary knee joint replacement substantial improvements in the scores for physical health, such as those for pain and physical functioning seem to take place within the first 3 to 6 months after surgery. Studies with longer-term follow-up describe long-lasting improvement [27]. Deehan et al. also found an enduring improvement in KSS at 3 and 12 months after revision surgery, which was comparable with the improvement in KSS for primary TKA. Successive surgical revision, however, had a negative influence on reported functional outcome [28]. In our series, after a median follow-up of 3.7 years, HSS, KSS and WOMAC scores showed inferior results for the patients who underwent TKA after tibial osteotomy; possibly due to the low numbers of patients these differences in function scores did not reach significance.

The last decade medial opening wedge HTO with special implants for internal fixation has gained popularity in the treatment of varus gonartrosis. Opening wedge osteotomy is advocated to be technically easier and a fibular osteotomy is not required. To our knowledge no results have yet been published on the effect on subsequent TKA. However, since larger implants are necessary in open wedge osteotomy, implant removal should not be combined with total knee arthroplasty. A recent RCT showed significantly more patellar descent and tibial slope increase after opening wedge HTO compared to the closing wedge technique [29]. This might cause exposure and patellar eversion problems during knee replacement. The advantage of opening wedge osteotomy is preservation of bone stock with tensioning of the medial collateral ligament. This may result in a more conservative amount of bone removed during knee joint replacement. Consequently, joint line elevation by the use of a thicker than desired tibial component in balancing the ligaments, is less likely. Furthermore, unlike closing wedge osteotomy, the relative position of the medullary canal is not altered. This may facilitate tibial component placement by intramedullary guidance. Future studies, however, have to confirm whether these aspects of the opening wedge osteotomy technique influence conversion to a TKA.

Correction osteotomy in relatively young patients with osteoarthritis of the medial compartment has good results and delays knee replacement [30]. Arthroplasty in the young patient can have adverse effects. In an update study of data from the Swedish Knee Arthroplasty Register younger age was associated with an increased risk of prosthetic revision [31]. An analysis of the Mayo Clinic total joint register confirms the significance of age in knee replacement. Ten-year knee prosthetic survivorship for patient who were 55 years or younger was 83% compared with 94% for those older than 70 years (p < 0.0001) [11]. It has been described that the survivorship for knee arthroplasties without prior surgery is significantly greater than for knees with prior operative treatment [11]. In our series, however, we did not encounter any TKA revisions because of loosening or infection in the group of patients receiving TKA after prior HTO during the course of our evaluation.

Ethical considerations, but also the absence of equipoise in performing randomisation due to the fact that outcomes of operations are related to their mechanisms of action as well as to patient's cooperation with treatment plans, rules out an RCT to assess the effects on TKA outcome after previous osteotomy [10]. A cohort study using comparison groups matched for patient-selection factors that affect survivorship of TKA will be the best available level of evidence. Our matched control study tends to show that total knee replacement after proximal tibial osteotomy is a therapeutic option technically more demanding than a primary knee arthroplasty. Medium-term results are less favourable for the osteotomized patient although not significantly worse. We are in need of more well-designed observational studies in order to conduct a meta-analysis to develop grades of recommendation in performing TKA after HTO.

## Competing interests

The author(s) declare that they have no competing interests.

## Authors' contributions

TR designed the study, examined the patients and drafted the manuscript. WB contributed substantially to the acquisition of data. MR carried out the statistical analysis and has been involved in interpretation of the data. JV participated in the study design and has been involved in critically revising the manuscript for important intellectual content. All authors read and approved of the final manuscript.

## Pre-publication history

The pre-publication history for this paper can be accessed here:


